# 4D Printing of Multi-Hydrogels Using Direct Ink Writing in a Supporting Viscous Liquid

**DOI:** 10.3390/mi10070433

**Published:** 2019-06-30

**Authors:** Takuya Uchida, Hiroaki Onoe

**Affiliations:** School of Integrated Design Engineering, Graduate School of Science and Technology, Keio University, 3-14-1 Hiyoshi, Kouhoku-ku, Yokohama, Kanagawa 223-8522, Japan

**Keywords:** 4D printing, 3D printing, stimuli-responsive hydrogel

## Abstract

We propose a method to print four-dimensional (4D) stimuli-responsive hydrogel structures with internal gaps. Our 4D structures are fabricated by printing an N-isopropylacrylamide-based stimuli-responsive pre-gel solution (NIPAM-based ink) and an acrylamide-based non-responsive pre-gel solution (AAM-based ink) in a supporting viscous liquid (carboxymethyl cellulose solution) and by polymerizing the printed structures using ultraviolet (UV) light irradiation. First, the printed ink position and width were investigated by varying various parameters. The position of the printed ink changed according to physical characteristics of the ink and supporting liquid and printing conditions including the flow rates of the ink and the nozzle diameter, position, and speed. The width of the printed ink was mainly influenced by the ink flow rate and the nozzle speed. Next, we confirmed the polymerization of the printed ink in the supporting viscous liquid, as well as its responsivity to thermal stimulation. The degree of polymerization became smaller, as the interval time was longer after printing. The polymerized ink shrunk or swelled repeatedly according to thermal stimulation. In addition, printing multi-hydrogels was demonstrated by using a nozzle attached to a Y shape connector, and the responsivity of the multi-hydrogels to thermal-stimulation was investigated. The pattern of the multi-hydrogels structure and its responsivity to thermal-stimulation were controlled by the flow ratio of the inks. Finally, various 4D structures including a rounded pattern, a spiral shape pattern, a cross point, and a multi-hydrogel pattern were fabricated, and their deformations in response to the stimuli were demonstrated.

## 1. Introduction

Accompanying the recent advances in three-dimensional (3D) printing technologies, not only static objects but also shape-changing structures have been fabricated by 3D printers using stimuli-responsive materials. These printed structures have been defined as four-dimensional (4D) printed structures by adding one dimension (time variation) to 3D printed structures [1,2,3,4,5]. 4D printed structures can change their shapes and functionalities in response to external stimuli such as light, heat, and pH changes. Thanks to these characteristics, 4D printed objects and machines can be expected to achieve self-assembly [6], self-adaptability [7], and self-repair [8]. 

In terms of the materials used for 4D printing, stimuli-responsive polymers [9] and hydrogels [10] have mainly been adopted. In particular, stimuli-responsive hydrogels have been applied to drug delivery systems [11] and soft actuators [12,13], owing to their biocompatibility and softness. Using stimuli-responsive hydrogels, 4D microstructures have usually been printed using photolithography [14,15,16,17,18] and deposition printing on substrates [19,20,21,22,23]. For all these methods, fabricated hydrogel structures are mainly layered structures, creating bending or twisting motions for the printed structures. However, it is difficult to fabricate 4D structures with internal gaps or suspended beam structures, both of which can be critical to achieving complex motions and encapsulating materials or micro-channels inside structures for soft robots or medical tools.

**Figure 1:**
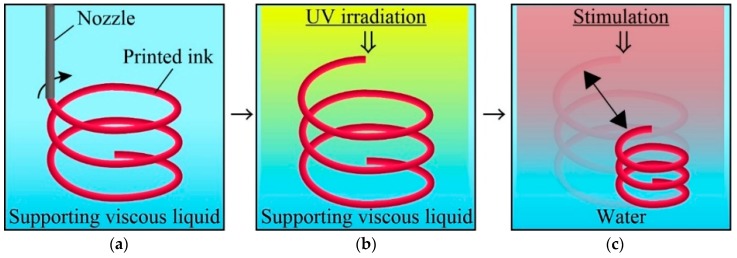
Concept of our proposed method for fabricating 4D structure with internal gaps. (**a**) Pre-gel monomer ink is ejected into supporting viscous liquid to print 3D ink pattern. (**b**) The printed ink is exposed to UV and is polymerized to a obtain 3D hydrogel structure. (**c**) After replaced the supporting liquid into water, the polymerized 3D hydrogel structure deforms in response to stimulation.

Here, we propose a new fabrication method for 4D printing that can fabricate 3D multi-hydrogels structures with internal gaps (Figure 1). We introduce a viscous liquid—carboxymethyl cellulose aq (CMC aq)—as a supporting viscous liquid during printing. As a printing ink, we chose a mixture of a poly-*N*-isopropylacrylamide (pNIPAM) solution, which exhibits a stimuli-responsive (thermo-responsive) shrinking/swelling characteristic after gelation, and a polyacrylamide (pAAM) solution, which does not respond to stimulation. To adjust the viscosity of the pNIPAM and pAAM print ink solution, we added a sodium alginate solution (NaAlg) to the printing ink. The print ink was printed directly through a nozzle in CMC aq (supporting viscous liquid) such that the printed ink can be maintained in the printed position to create 3D patterns with internal gaps. Then, the printed 3D ink patterns can be polymerized using ultraviolet (UV) irradiation. We printed a straight line of ink and investigated the position and width of the printed ink under various conditions. Next, we printed a corner of the ink and investigated the printing resolution. We polymerized the printed ink in the supporting viscous liquid and investigated the degree of gelation and the responsivity to external stimuli. In addition, we polymerized multi-hydrogels and investigated their printed pattern and responsivity to stimuli. Finally, we printed various 4D structures and investigated their responsivity to thermal stimuli. Our method can provide an effective tool for fabricating hydrogel 4D structures with various types of physical or chemical stimuli for applications in soft actuators/robotics and self-assembly/adaptive systems. 

## 2. Materials and Methods 

### 2.1. Materials

*N*-isopropylacrylamide (NIPAM) (monomer) (113.16 g/mol, 095-03692) and *N*,*N*′-methylene-bis-acrylamide (BIS) (cross-linking agent) (154.17 g/mol, 134-02352) were purchased from FUJIFILM Wako Chemicals USA, Corp. (Richmond, VA, USA). IRGACURE1173 (photo polymerization initiator) was purchased from BadischAnilin and Soda-Fabrik (Ludwigshafen, Germany). In addition, sodium alginate (NaAlg) (80–120 cp, 194-13321) and acrylamide (AAM) (71.08 g/mol, 019-08011) were purchased from FUJIFILM Wako Chemicals USA, Corp. (Richmond, VA, USA), and carboxymethyl cellulose (CMC) (1000–50000 Pa·s, CMF-150) was purchased from AS ONE Corporation (Osaka, Japan). New Coccine (coloring dye) was purchased from Kyoritsusyokuhin Inc. (Osaka, Japan), and acryloxyethyl thiocarbamoyl rhodamine B (652.2 g/mol, 25404-100) was purchased from Polyscience, Inc. (Warrington, PA, USA). Fluorescence beads (1934417A, 1927586) were purchased from Thermo Fisher Scientific (Waltham, MA, USA). All chemicals were utilized with no further purification. Deionized water was obtained from a Millipore purification system. Table 1 shows a glossary of the abbreviation of materials.

### 2.2. Set Up for 4D Printing

**Figure 2:**
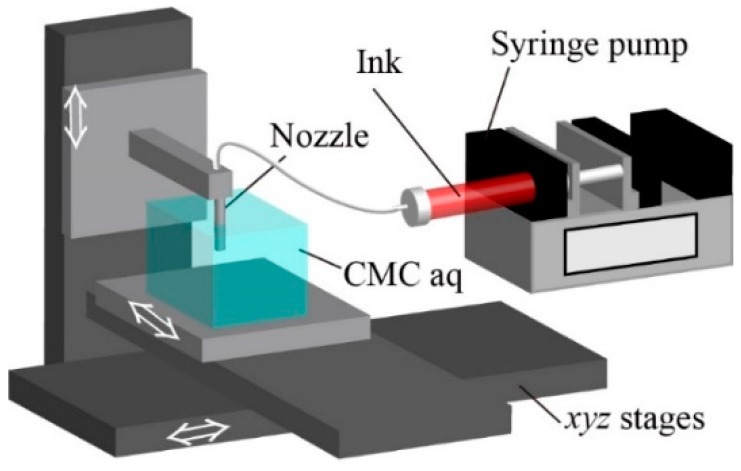
Schematic of setup. A nozzle is fixed on a *z* stage, and a container of carboxymethyl cellulose aq (CMC aq) is fixed on *xy* stages. By moving the *xyz* stages, the pre-gel ink is ejected via the nozzle into CMC aq by a syringe pump.

The ink was injected using a syringe pump (LEGATO 180, KD Scientific, Holliston, MA, USA) through a nozzle composed of SUS304 (NN-2225R, TERUMO, Tokyo, Japan) in CMC aq, using *xyz* stages (OSMS20-(XY), OSMS26-(Z), SIGMAKOKI, Tokyo, Japan). The nozzle was fixed with a jig. The program of the stages was set using sample103 (SIGMAKOKI). Figure 2 illustrates the setup for our printing system.

### 2.3. Evaluation of Printed Ink Patterns in Supporting Material

The printing ink for our 4D printing was composed of 10% (*w/w*) NIPAM monomer, 0.01% (*w/w*) BIS, 1% (*w/w*) New Coccine, 0.5% (*w/w*) IRGACURE1173, and 1–3% (*w/w*) NaAlg. The supporting viscous liquid, 0.4–1.6% (*w/w*) CMC was tested (concentration of CMC: *C_CMC_*). To investigate printing performance, we printed a straight line (20 mm in length) of printing ink under various conditions, as follows (Table 2). The flow rate of the printing ink, *Q*, was 0.5–1.5 µL/s, and the stage speed, *v*, was 0.5–1.5 mm/s. In addition, the diameter and depth of the nozzle, *d* and *h*, were 400–800 µm and 5–10 mm, respectively. Table 2 shows the values of the all parameters.

To evaluate the corner patterns, a 20 mm line with a single corner (corner angle *θ*: 30–150°) was printed (ink: 10% (*w/w*) NIPAM monomer, 0.01% (*w/w*) BIS, 1% (*w/w*) New Coccine, and 3% (*w/w*) NaAlg. Conditions: *C_CMC_* = 1% (*w/w*), *v* = 1.0 mm/s, *Q* = 1.0 µL/s, *d* = 400 µm, and *h* = 5 mm) and analyzed. All printing was independently conducted three times. The printed ink was captured when printing using a microscope (VH-5500, KEYENCE, Osaka, Japan) from the *z*-axis and *y*-axis.

In all experiments we conducted, we used symbols defined in Table 3. The detailed definitions of these symbols are described in each experiment section.

### 2.4. Polymerization of Printed Ink in Supporting Viscous Liquid

**Figure 3:**
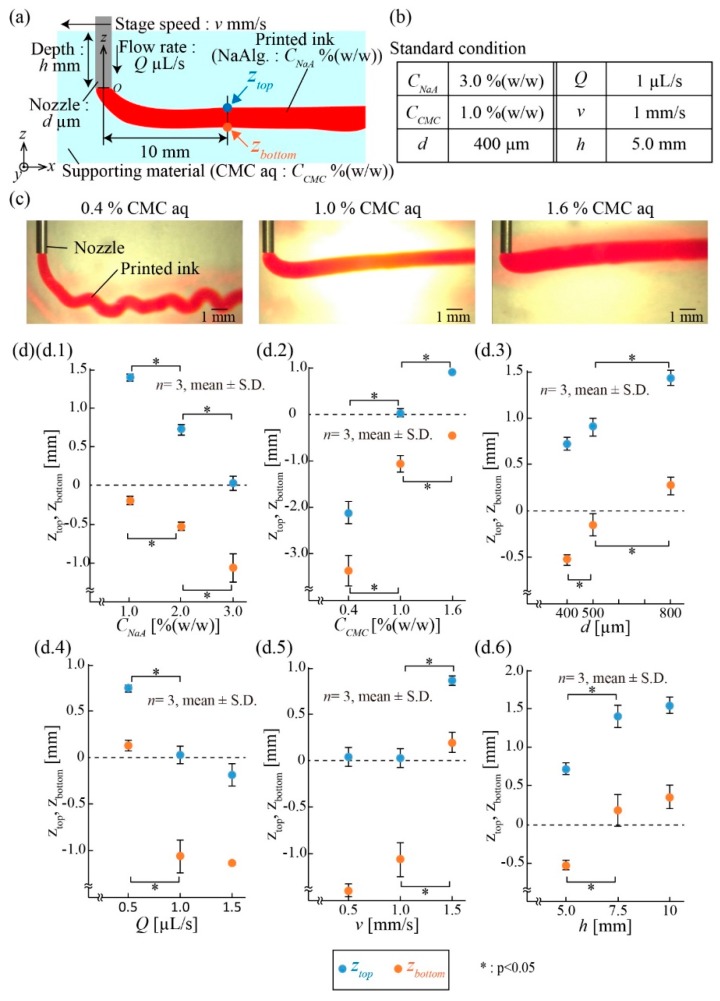
Measurement of the position of the printed ink in the supporting viscous liquid. (**a**) Schematic of *z_top_* and *z_bottom_* for the printed ink and parameters. (**b**) Table of the standard conditions. (**c**) Images of the printed ink with various concentrations of CMC aq. The *z_top_* and *z_bottom_* increased when the concentration of CMC aq increased. (**d**) The *z_top_* and *z_bottom_* for the printed ink under various conditions. The dotted line shows the position of the tip of the nozzle.

Acryloxyethyl thiocarbamoyl rhodamine B (0.002% (*w/w*)) was added to the printing ink to visualize the polymerized hydrogel. After printing a 20 mm straight line of the ink under the standard printing condition (Figure 3b: *C_CMC_* = 1% (*w/w*), *v* = 1.0 mm/s, *Q* = 1.0 µL/s, *d* = 400 µm, and *h* = 5 mm), the printed ink was exposed to UV light (170 mW/cm^2^, HLR100T-2, SEN LIGHTS CORPORATION, Osaka, Japan) at an interval time of 20–60 s after printing. After the irradiation, the container of CMC aq was placed into a beaker filled with water to replace CMC with the water to obtain the polymerized ink. The polymerized ink was placed into water that had settled at room temperature, imaged using a fluorescence microscope (IX73P1-22FL/PH, OLYMPUS, Tokyo, Japan), and measured using imaging software (Cellsens, OLYMPUS). Then, we obtained the gelation ratio (*d_polymer_/d_initial_*, where *d_polymer_* is the diameter of the polymerized ink and *d_initial_* is the width of the printed ink).

### 2.5. Responsivity of Polymerized Printed Ink

When heating, the polymerized printed ink was placed into heated water (48 °C), which was heated to the specific temperature using a hotplate (ND-1, AS ONE). The temperature of the water was measured using a thermometer (HI98501, Hanna Instruments, Woonsocket, RI, USA). When cooling, the polymerized printed ink was placed into water that had settled at room temperature. The heated or cooled polymerized ink was imaged using a fluorescence microscope and measured using the Cellsens software. Then, we obtained the shrinking ratio (*w_n_*/*w*_0_, where *w_n_* is the diameter of the heated or cooled polymerized ink and *w*_0_ is the initial diameter of the polymerized printed ink).

### 2.6. Printing of Multi-Hydrogels Structures

We printed multi-hydrogels, including NIPAM-based and AAM-based ink. The NIPAM-based ink was composed of 10% (*w/w*) NIPAM, 0.02% (*w/w*) BIS, 3% (*w/w*) NaAlg, and 1% (*w/w*) fluorescence beads. The AAM-based ink was composed of 10% (*w/w*) acrylamide, 0.02% (*w/w*) BIS, 1% (*w/w*) fluorescence beads, 0.5% (*w/w*) IRGACURE1173, and 3% (*w/w*) NaAlg. We attached a Y-shaped connecter to the nozzle. We printed multi-hydrogels simultaneously, where the flow rate of the NIPAM-based ink was 0.5–0.7 µL/s, and the flow rate of the AAM-based ink was 0.3–0.5 µL/s. UV light was irradiated on the printed ink for 60 s. After the irradiation, the container of CMC aq was placed into a beaker filled with water to get the multi-hydrogel. Then, the multi-hydrogels were placed into heated water (48 °C) for 5 min. We obtained the curvature using a microscope.

### 2.7. Demonstration of 4D Printing

The NIPAM-based ink was composed of 10% (*w/w*) NIPAM monomer, 0.01% (*w/w*) BIS, 1% (*w/w*) fluorescence beads, 0.5% (*w/w*) IRGACURE1173, and 3% (*w/w*) NaAlg. The AAM-based ink was composed of 10% (*w/w*) acrylamide, 0.02% (*w/w*) BIS, 1% (*w/w*) fluorescence beads, 0.5% (*w/w*) IRGACURE1173, and 3% (*w/w*) NaAlg. We printed a circle, the character “T”, and a spring shape using the NIPAM-based ink only. We also printed the character “C” by ejecting NIPAM-based and AAM-based ink. The printed structures were exposed to UV light for 60 s to polymerize. After irradiation, the container of CMC aq was placed into a beaker filled with water to get the polymerized structures. Then, the polymerized structures were placed into water that had settled at room temperature. Then, the polymerized structures were placed into heated water (48 °C), which was heated using a hotplate. The polymerized structures were observed using a microscope.

## 3. Results

### 3.1. Position of Printed Ink in Supporting Viscous Liquid

Our printing system was simply composed of a nozzle consisting of a needle, a syringe pump, and *xyz*-motorized precision stages (Figure 2). The printed ink was ejected from the nozzle into a container filled with CMC aq and kept the constant width of the printed pattern. In our system, CMC aq was viscous (8.11–383 mPa·s) and worked as a supporting viscous liquid for the printed ink, enabling us to fabricate 3D hydrogel structures after the polymerization of the patterned ink.

To investigate the printing performance of the ink in CMC aq, we evaluated the printed position of the ink. We printed a straight 20 mm line of ink and measured the top and bottom positions (*z*-axis) of it, *z*_top_ and *z*_bottom_, respectively, at 10 mm from the nozzle (Figure 3a). We tested six parameters: Two liquid parameters (the concentrations of NaAlg in the printed ink *C_NaA_* and CMC in the supporting viscous liquid *C_CMC_*), two ejection parameters (the diameter *d* of the nozzle and the flow rate *Q* of the ejected ink solution), and two printing parameters (the stage speed *v* and the depth of the nozzle *h*). The listed values for these parameters (Figure 3b) were set as a standard condition for the experiment. These parameters were varied one at a time to examine the effect on the printed ink pattern. For example, Figure 3c presents the images of printed ink patterns in the *z*-axis when *C_CMC_* was varied as 0.4%, 1.0%, and 1.6%. 

Figure 3d.1–d.6 shows the plots of *z*_top_ and *z*_bottom_ when single parameters were varied. For the liquid parameters, *z*_top_ and *z*_bottom_ increased when *C_NaA_* decreased or *C_CMC_* increased (Figure 3d.1,d.2). As these concentrations determine the viscosities of the liquids, the results suggest that the *z* position of the printed ink can be adjusted through the viscosities of the printed ink and supporting fluid. For the ejection parameters, *z*_top_ and *z*_bottom_ increased when *d* increased or *Q* decreased (Figure 3d.3,d.4), indicating that the *z* position of the ink depends on the ejection speed, *V*, of the ink at the nozzle tip, described as.

(1) $$V=\frac{Q}{\pi {\left(\frac{d}{2}\right)}^{2}}$$

Finally, for the printing parameters, the *z*_top_ and *z*_bottom_ increased when *v* or *h* increased (Figure 3d.5,d.6). We consider that pressure loss occurred at the back of the cylindrical nozzle, where the nozzle moved. Because pressure loss depends on *v* and *h*, the position of the printed ink was moved upward.

### 3.2. Width of Printed Ink in Supporting Viscous Liquid

**Figure 4:**
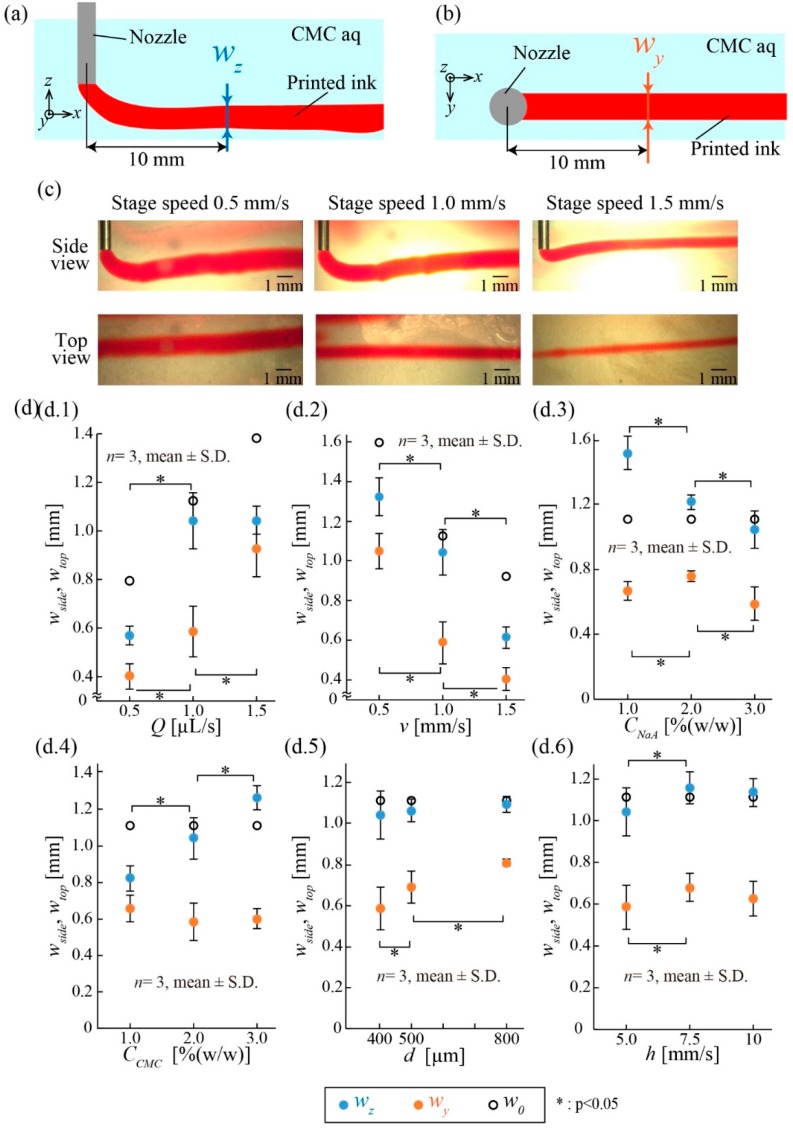
Measurement of the width of the printed ink in the supporting viscous liquid. (**a**) *w_z_* is defined as the *z*-axis width of the printed ink at 10 mm away from the nozzle. (**b**) *w_y_* is defined as the *y*-axis width of printed ink at 10 mm away from the nozzle. (**c**) Images of the printed ink with various stage speeds. *w_z_* and *w_y_* decreased as the stage speed increased. (**d**) *w_z_* and *w_y_* under various conditions. *w_z_* and *w_y_* can be mainly controlled by the flow rate and stage speed.

Next, we investigated the width of the printed ink in terms of the above parameters. Similarly to the experiment in 3.1, we printed a 20 mm straight line of ink and measured the width of the printed ink from the side view (*z*-axis width), *w*_z_, and top view (*y*-axis width), *w*_y_, at 10 mm away from the nozzle (Figure 4a,b). Based on the standard conditions in Figure 3b, the parameters were varied one at a time. For example, Figure 4c shows that the widths, *w*_z_ and *w_y_,* of the printed ink patterns changed at stage speeds *v* of 0.5, 1.0, and 1.5 mm/s. 

Figure 4d.1–d.6 presents the plots of *w*_z_ and *w*_y_ when individual parameters were varied. In our experiments, *w*_z_ and *w*_y_ approximately ranged from 600 µm to 1.3 mm and 400 µm to 1 mm, respectively. In addition to these experimental values, the theoretical width of the printed ink, *w*_0_, expressed as

(2) $$w_{0}=\sqrt{\frac{Q}{v{\left(\frac{d}{2}\right)}^{2}}}$$

was also plotted as open circles. Note that we hypothesized that the shape of the printed ink was an ideal cylinder. 

The widths of the printed ink, *w*_z_ and *w*_y_, varied depending on *Q* and *v* (Figure 4d.1,d.2). Both widths increased when *Q* increased or *v* decreased. This tendency matched with the theoretical description in Equation (1). For the liquid parameters, *w_z_* decreased as *C_NaA_* increased, although *w_y_* remained constant (Figure 4d.3). Furthermore, *w_z_* increased as *C_CMC_* increased, although *w_y_* also remained constant (Figure 4d.4). We consider that the changes in *w_z_* resulted from the drag force in the *z*-direction caused by the pressure loss around the nozzle. The two parameters *d* and *h* did not influence *w*_z_ and *w*_y_ (Figure 4d.5,d.6). 

The tendencies of the position and width of the printed ink when the parameters varied are summarized in Table 4. The results confirm that, although the *z*-axis position was affected by the various parameters, the width of the printed ink could be simply controlled by adjusting *Q* and *v*. Unless otherwise noted, we adopted the standard conditions (Figure 3b) for the following printing experiments. 

### 3.3. Evaluation of Printed Ink Patterns

**Figure 5:**
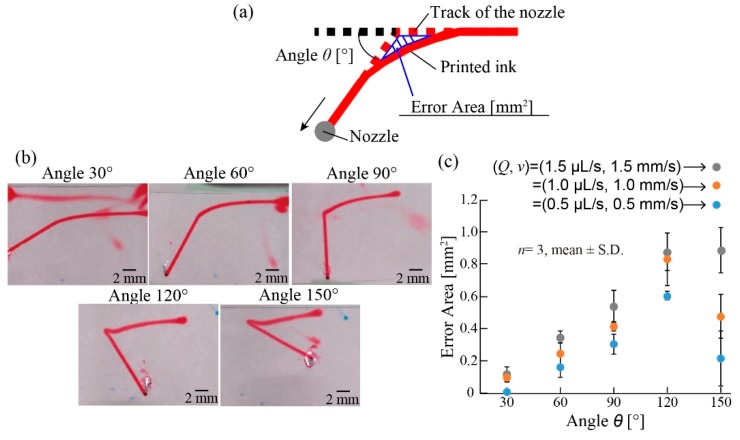
Evaluation of printed ink pattern. (**a**) The error area is defined as the dragged area of the patterned ink at the corner. (**b**) Images of the printed corners with different angles *θ*. The patterned ink (*v* = 1.0 mm/s, *Q* = 1.0 µL/s) was distorted depending on the angle of the corner. (**c**) Relationship between the error area and *θ* with different *Q* and *v* values. The error area increased as the angle increased, reaching the maximum when the angle was 120° for all three nozzle speeds. For all angles, the slower the nozzle speed, the lower the error area.

The printed inks can be patterned in the supporting liquid using programmed motions of the *x*, *y*, and *z* motors. To examine drawing capability, we printed a line with a single corner of various angles *θ* ranging from 30 to 150° (Figure 5). Ideally, the patterns of the printed ink should be the same as the tracks of the nozzle. However, the patterns of the printed ink were slightly dragged by the motion of the nozzle and did not exactly match with the tracks of the nozzle (Figure 5a). To evaluate the difference between the printed patterns and the track of the nozzle, we defined the error area *A*_error_ as the dragged area of the patterned ink at the corner (Figure 5a).

As shown in Figure 5b, the patterned ink (*v* = 1.0 mm/s, *Q* = 1.0 µL/s) was distorted depending on the angle of the corner. We examined the relationship between the error area *A*_error_ and corner angle *θ* for three different nozzle speeds and flow rates (*v* = 0.5, 1.0, and 1.5 mm/s: *Q* = 0.5, 1.0, and 1.5 µL/s, respectively) to keep the diameter of the printed ink patterns constant. The error area *A*_error_ increased as the angle *θ* increased, reaching the maximum when *θ* was 120° for all three nozzle speeds (Figure 5c). For all angles *θ*, the slower the nozzle speed *v*, the lower the error area *A*_error_. According to these results, to print a corner pattern precisely, a small flow rate and slow stage speed should be chosen.

### 3.4. Polymerization of Printed Ink in Supporting Viscous Liquid

**Figure 6:**
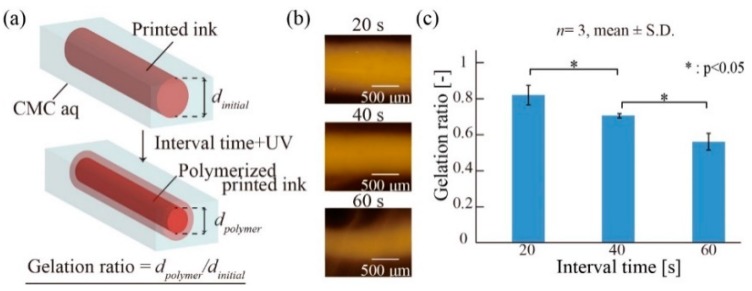
Polymerization of the printed ink in CMC aq. (**a**) Gelation ratio is defined as the diameter of the polymerized ink after UV irradiation divided by the diameter of the printed ink pattern before UV irradiation. (**b**) Fluorescence images of the polymerized printed ink. (**c**) Gelation ratio for different interval times. The gelation ratio decreased as interval time increased.

In our printing method, the printed ink is polymerized using UV irradiation in the supporting viscous liquid (CMC aq), where the printed ink gradually diffuses. Thus, the interval time between the UV irradiation and printing is important for polymerization. We examined the relationship between the polymerization of the printed ink in CMC aq and the interval time. To verify the polymerization, we defined the gelation ratio as the diameter *d*_polymer_ of the polymerized pattern after the UV irradiation divided by the diameter *d*_initial_ of the printed ink pattern before UV irradiation (Figure 6a). Figure 6b shows that the diameter of the polymerized ink became narrower as the interval time increased. Thus, the gelation ratio decreased as the interval time increased (Figure 6c). This is because the gelation area became narrower, owing to the diffusion of the printed ink in CMC aq before UV irradiation. These results indicate that a shorter interval time between printing and UV irradiation can reduce the difference between the polymerized patterns and printed ink patterns.

### 3.5. Responsivity of External Stimuli

**Figure 7:**
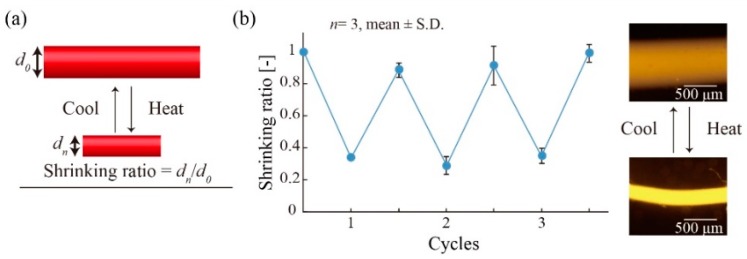
Responsivity of the polymerized hydrogel to thermal stimuli. (**a**) Definition of the shrinking ratio *d_n_/d_0_*. Shrinking ratio is obtained by the diameter of the polymerized printed ink after being heated or cooled divided by the initial diameter of the polymerized printed ink before stimuli. (**b**) The repeatability of shrinking/swelling behavior of the polymerized printed ink. The polymerized printed ink shrunk or expanded according to heating or cooling.

We investigated the responsivity of the polymerized printed hydrogel patterns to thermal stimulation. The shrinking/swelling characteristics of the polymerized hydrogel pattern were evaluated by measuring the shrinking ratio *w_n_*/*w*_0_ in response to changes in the temperature (Figure 7a), where *w*_0_ is the initial width of the polymerized pattern, *w_n_* is the width of the pattern after stimulation, and *n* is the number of stimuli. We repeatedly heated (48 °C) and cooled (23 °C) the polymerized hydrogel pattern (5 min per each cycle) and evaluated the shrinking ratio (Figure 7b). The shrinking ratio was repeatedly varied from approximately 0.3 to 0.95, corresponding to heating and cooling, respectively. These results suggest that the polymerized printed hydrogel patterns could deform repeatedly in response to heating/cooling cycles.

### 3.6. Printing of Multi-Hydrogels Structures

**Figure 8:**
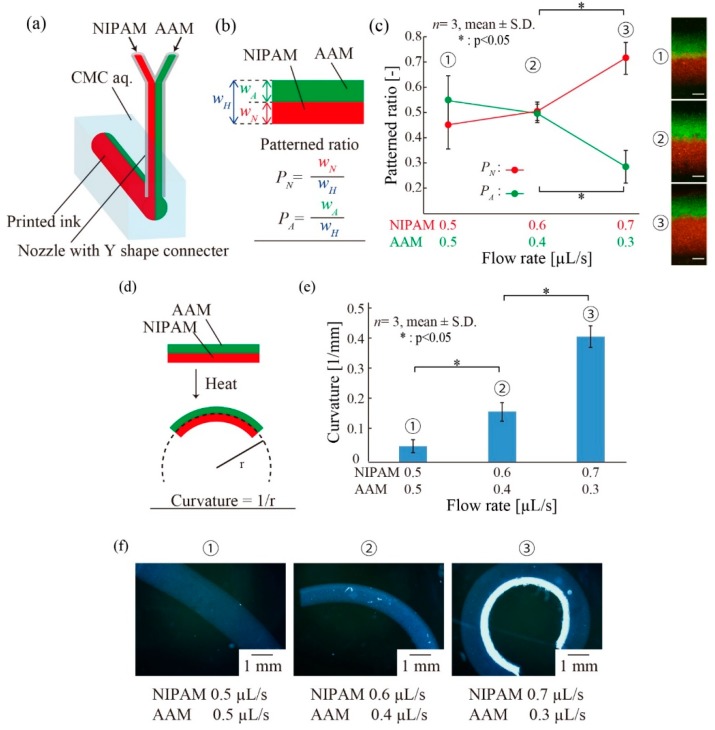
Printing of multi-hydrogel structures. (**a**) A schematic of the setup to print multi-hydrogel inks. The N-isopropylacrylamide-based stimuli-responsive pre-gel solution (NIPAM)-based pre-gel ink and the acrylamide-based non-responsive pre-gel solution (AAM)-based pre-gel ink were ejected into CMC aq via the nozzle attached to a Y shape connector. (**b**) The definition of the patterned ratios *P_N_* and *P_A_* which is obtained by the width of poly-*N*-isopropylacrylamide (pNIPAM) gel or polyacrylamide (pAAM) gel divided by the total width of the polymerized inks, respectively. (**c**) Results for the patterned ratios *P_N_* and *P_A_* with different flow rates *Q.* The scale bars are 500 µm. The *P_N_* increased and the *P_A_* decreased when the flow rate of the NIPAM ink increased and that of the AAM ink decreased. (**d**) The definition of the curvature radius of a heated multi-hydrogels structure. (**e**) Results for the curvature radius of heated multi-hydrogels structures with different flow rates. The curvature increased when the flow rate of the NIPAM-based ink increased. (**f**) Images of the deformed multi-hydrogels structures.

In addition to investigating the simple shrinking/swelling motions of the printed hydrogel patterns, the printing of multiple types of materials was performed, enabling various deformations. We utilized two different pre-gel inks, NIPAM-based and AAM-based, and infused these inks to the nozzle simultaneously via a Y-shaped connecter (Figure 8a). After UV irradiation, the polymerized hydrogel pattern exhibited a double-layer structure. We prepared three different double-layer hydrogel structures which were fabricated by NIPAM/AAM flow rates of 0.5/0.5 µL/s, 0.6/0.4 µL/s, and 0.7/0.3 µL/s. First, we investigated the printing pattern of the double-layer structure by measuring the patterned ratios *P_N_* and *P_A_* (*P_N_ = w_N_/w_H_*, *P_A_* = *w_A_/w_H_*, where *w_N_* is the width of the pNIPAM gel, *w_A_* is the width of the pAAM gel, and *w_H_* is the total width) (Figure 8b). Figure 8c shows that *P_N_* increased and *P_A_* decreased when the flow rate of the NIPAM ink increased and that of the AAM ink decreased. Next, we investigated the responsivity of the double-layer structure to a thermal stimulus. The pNIPAM hydrogel responds to thermal stimuli, whereas the pAAM hydrogel does not. Thus, a folding motion of the polymerized double-layer hydrogel pattern can be achieved in response to a thermal stimulus. We measured the curvatures of these double-layer hydrogel patterns after heating (Figure 8d). The curvature of the double-layer hydrogel structure increased when the flow rate of the NIPAM-based ink increased (Figure 8e,f). A large curvature resulted from increasing the cross-sectional area of the pNIPAM. Figure 8c,e shows that a large curvature can be achieved by setting a large flow rate for the NIPAM ink to increase *P_N_*. These results suggest that deformation control can be achieved by printing multi-hydrogels with controlled layer thicknesses by adjusting the flow rate of the ink.

### 3.7. Demonstration of 4D Printing

**Figure 9:**
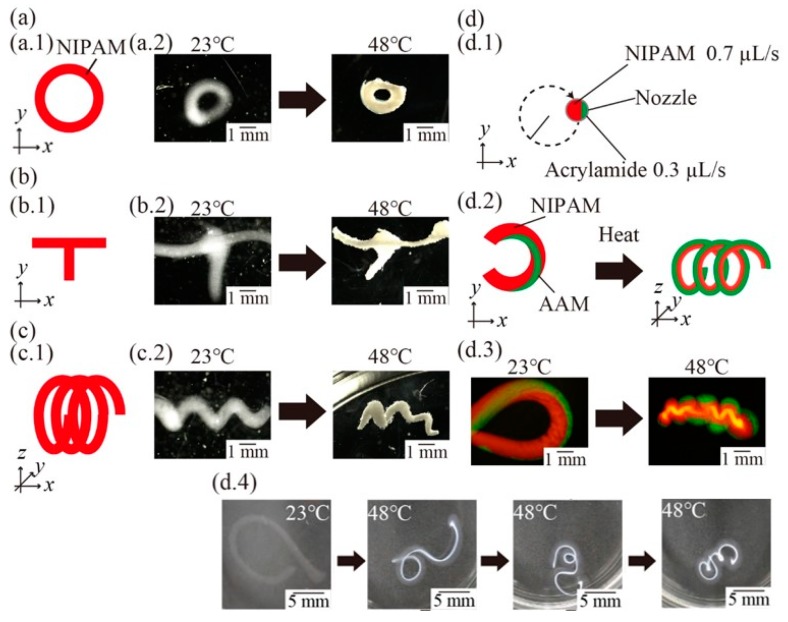
A demonstration of 4D printing. (**a**) Schematic illustration and images of printed structures and heated structures with a rounded pattern. (**b**) Schematic illustration and images of a fabricated T-shaped structure and a heated T-shaped structure with a cross point. (**c**) Schematic illustration and images of a fabricated spring structure and heated spring structure with an internal gap. (**d**) Schematic illustrations of printing a C-shaped structure with multi-hydrogels (**d.1**) and its 3D deformation from the C-shaped structure to the spring-shaped structure. (**d.2**) Fluorescence images of the fabricated C-shaped structure (**d.3**) and the transformed spring structure obtained by heating. (**d.4**) Time-lapse images of the C-shaped structure.

Finally, we fabricated variations of 4D structures to demonstrate the effectiveness of the proposed method. We set the program of stages to be performed in a circle (Figure 9a.1). Figure 9a.2 presents the images of the fabricated circular structure and its response to thermal stimuli, indicating that not only angulated patterns (see Section 3.3) but also rounded patterns can be fabricated using our method. Furthermore, we printed the character “T” and a 3D spring shape (Figure 9b.1,c.1). Figure 9b.2,c.2 presents the images of the fabricated structures and their response structures, indicating that the cross point (Figure 9b.2) and internal gaps (Figure 9c.2) can be fabricated and maintained following stimulation. By printing cross points, complex 4D structures such as deformable 3D meshes could be fabricated. 

Finally, we printed the character “C” by moving the stages in a “C” shape and ejecting NIPAM-based (0.7 µL/s) and AAM-based (0.3 µL/s) ink at the same time (Figure 9d.1). The printed structure exhibited twisted ink patterns and deformed three-dimensionally into a spiral shape (Figure 9d.2). Figure 9d.3 presents the images of the fabricated structure, a spring shape, and its response. Figure 9d.4 presents the time-lapse images of the deformation. Figure 9d.3,d.4 shows that the fabricated 4D structure can deform three-dimensionally by printing with multi-hydrogels. By printing internal gaps and multi-hydrogels, 4D structures can exhibit various deformations in response to external stimuli, as there is space in which to deform. These results suggest that our proposed method could enable the printing of complex 4D multi-hydrogels structures with cross points and internal gaps.

## 4. Discussion

We demonstrated a fabrication method for 4D structures composed of multiple types of stimuli-responsive hydrogels while using a viscous liquid as the supporting material during printing with the simple setup: A nozzle, syringe pumps, and motorized stages. Regarding printing performance, the condition shown in Figure 3b is suitable for printing conditions in the current setup. The width of the printed ink is controlled by *Q* and *v* rather than the diameter of the nozzle *d*. In addition, our approach allows us to expand the number of printing materials by using a nozzle with branched channels. Regarding printable structures in our method, it is possible to fabricate 4D structures with internal gaps that have not been fabricated in previous methods [10,11]. By printing internal gaps, it is possible to create functional materials and machines that achieve complex motions or material encapsulation inside the printed structures. Regarding the deformation of 4D structures in response to stimuli with our method, various complex motions of printed structures could be achieved depending on the patterned ratio of multi-layered structures controlled by an adequate flow ratio of NIPAM/AAM inks. In addition, 4D structures with internal gaps can deform in various ways, including the formation of 3D spring structures from a 2D printed C-shaped pattern (Figure 9d) and the anisotropic deformation of multi-layered stimuli-responsive hydrogel springs [24]. Moreover, those 4D structures could deform repeatedly in response to thermal-stimuli because the repeatable responsivity of pNIPAM gel to thermal-stimuli was already reported elsewhere [25,26]. During deformation, the delamination of gel at the cross point (Figure 9b) did not occur (even if the area of the bonding area was smaller than the width of the printed ink) because of proper crosslinking by covalent bond between the layers (c.f. three possible combinations of layers: pNIPAM/pNIPAM gel layers, pNIPAM/pAAM gel layers, and pAAM/pAAM gel layers) [12,27]. 

Regarding 4D printing techniques, printing ink directly in a viscous liquid is characteristic in our printing method. On the other hand, similar methods in 3D printing techniques have been demonstrated: Printing ink directly in a supporting self-healing gel to fabricate hydrogel 3D structures [28], electronics [29], and vascular networks [30] has been reported. In addition, printing ink directly in granular gel to fabricate silicon 3D structures and hydrogel 3D structures [31] has been reported. Compared to those reported methods, we used a simple viscous supporting material, CMC aq. That is, the printing performance of our method could be enhanced by combining it with reported methods.

There are some challenges in this method. First, the printed ink in the supporting viscous liquid was dragged in the direction of movement of the nozzle because the flow of the supporting viscous liquid (CMC aq) occurred around the nozzle. To improve this, it is necessary to reduce the flow of the supporting material by using a nozzle with a small diameter or by decreasing the stage speed. Second, the printed ink becomes narrow and gradually disappears because of its diffusion in the supporting viscous liquid (Figure 6c). This phenomenon would be a problem for printing large-scale patterns that take longer to print. To solve this problem, it is necessary to polymerize the printed ink immediately after the ink is printed in the supporting viscous liquid. For example, an integrated nozzle that has an optical waveguide for UV irradiation at the tip of the nozzle could be adopted for immediate polymerization.

## 5. Conclusions

We proposed a new fabrication method for 4D structures composed of stimuli-responsive hydrogels by using a viscous liquid as a supporting material during printing. Using this method, printed 4D structures with internal gaps, which have not been fabricated using previous methods, can be fabricated. We confirmed that the position of the printed ink was influenced by various parameters. The widths of the printed ink, *w*_z_ and *w_y_*, ranged from approximately 600 µm to 1.3 mm and 400 µm to 1 mm, respectively. To print 4D structures accurately, a slower stage speed and smaller nozzle diameter should be utilized. Polymerized printed ink that has been irradiated by UV in liquid CMC aq can repeatedly respond to external stimulation. Complex 4D structures exhibiting various deformations in response to external stimuli could be fabricated by printing multi-hydrogels with cross points and internal gaps. We believe that our proposed method would be useful for printing complex 4D structures with multiple functions in environmental monitoring and medical applications.

## Figures and Tables

**Table 1:** Glossary of abbreviation of materials.

Material	Abbreviation
*N*-isopropylacrylamide	NIPAM
carboxymethyl cellulose	CMC
Acrylamide	AAM
sodium alginate	NaAlg

**Table 2:** Value of the all parameters.

Parameter	Value	Unit
Concentration of NaAlg, *C_NaA_*	1.0, 2.0, 3.0	% (*w/w*)
Concentration of CMC, *C_CMC_*	0.4, 1.0, 1.6	% (*w/w*)
Flow rate of a syringe pump, *Q*	0.5, 1.0, 1.5	µL/s
Stage speed, *v*	0.5, 1.0, 1.5	mm/s
Diameter of a nozzle, *d*	400, 500, 800	µm
Depth of a nozzle, *h*	5.0, 7.5, 10	mm

**Table 3:** Glossary of symbols.

Define	Symbol
Maximum position of printed ink	*z_top_*
Minimum position of printed ink	*z_bottom_*
*z*-axis width of printed ink	*w_z_*
*y*-axis width of printed ink	*w_y_*
Dragged area of the patterned ink at the corner	*A_error_*
Diameter of polymerized ink	*d_polymer_*
Diameter of printed ink	*d_initial_*
Diameter of polymerized ink before stimuli	*d_0_*
Diameter of polymerized ink after stimuli	*d_n_*
Patterned ratio of pAAM gel in multi-hydrogel structure	*P_A_*
Patterned ratio of pNIPAMgel in multi-hydrogel structure	*P_N_*
Width of pAAM gel in multi-hydrogel structure	*w_A_*
Width of pNIPAM gel in multi-hydrogel structure	*w_N_*
Total width of multi-hydrogel structure	*w_H_*

**Table 4:** Trends of *z_top_*, *z_bottom_*, *w_y_*, and *w_z_* under printing parameter changes.

Parameter	*z_top_, z_bottom_*	*w_z_*	*w_y_*
*C_NaA_*↑	↓	↑	→
*C_CMC_*↑	↑	↓	→
*d*↑	↑	→	
*Q*↑	↓	↑	
*v*↑	↑	↓	
*h*↑	↑	→	
